# Supplementary material for: Assessing Quality of Life in Hemodialysis Patients in Kazakhstan: A Cross-Sectional Study
Source: J Clin Med. 2025 Jul 16;14(14):5021. doi: 10.3390/jcm14145021 (PMC12295577; doi:10.3390/jcm14145021)
Supplement: Supplementary file 1 [file jcm-14-05021-s001.zip › jcm-3707559-supplementary tables S2-S5, figure S1.pdf]

Supplementary Materials

Suppl. Table S2. Distribution of ESRD etiology by dialysis duration (χ^2=27.8, p=0.268)

**ESRD etiology**	**n**	**Dialysis duration**				
		**< 1 year**	**1-3 years**	**4-6 years**	**7-10 years**	**> 10 years**
Glomerulonephritis	67	10 (14.9%)	21 (31.3%)	10 (14.9%)	15 (22.4%)	11 (16.4%)
Diabetes mellitus	30	5 (16.7%)	9 (30.0%)	10 (33.3%)	4 (13.3%)	2 (6.7%)
Arterial hypertension	23	6 (26.1%)	8 (34.8%)	6 (26.1%)	2 (8.7%)	1 (4.3%)
Polycystic kidney disease	20	6 (30.0%)	6 (30.0%)	6 (30.0%)	0	2 (10.0%)
Pyelonephritis	14	0	5 (35.7%)	5 (35.7%)	2 (14.3%)	2 (14.3%)
Other	34	6 (17.6%)	10 (29.4%)	8 (23.5%)	4 (11.8%)	6 (17.6%)
Don't know	29	8 (27.6%)	12 (41.4%)	4 (13.8%)	5 (17.2%)	0

Suppl. Table S3. Bayesian ANOVA assessing the effect of ESRD etiology, dialysis duration, and their interaction on Kidney Disease Component Summary

**Models**	**P(M)**	**P(M|data)**	**BF_M**	**BF_{10}**	**Error%**
Null model	0.200	0.224	1.157	1.000	
ESRD etiology	0.200	0.110	0.484	0.481	0.0001
Dialysis duration	0.200	0.361	2.265	1.611	0.0003
ESRD etiology + Dialysis duration	0.200	0.238	1.247	1.059	2.990
ESRD etiology + Dialysis duration + ESRD etiology * Dialysis duration	0.200	0.068	0.294	0.305	0.600

Suppl. Table S4. Bayesian ANOVA assessing the effect of ESRD etiology, dialysis duration, and their interaction on Physical Component Summary

**Models**	**P(M)**	**P(M|data)**	**BF_M**	**BF_{10}**	**Error%**
Null model	0.200	0.756	12.366	1.000	
ESRD etiology	0.200	0.042	0.175	0.055	0.0034
Dialysis duration	0.200	0.189	0.931	0.250	0.00007
ESRD etiology + Dialysis duration	0.200	0.012	0.049	0.016	0.489
ESRD etiology + Dialysis duration + ESRD etiology * Dialysis duration	0.200	0.002	0.007	0.002	0.688

Suppl. Table S5. Bayesian ANOVA assessing the effect of ESRD etiology, dialysis duration, and their interaction on Mental Component Summary

**Models**	**P(M)**	**P(M|data)**	**BF_M**	**BF_{10}**	**Error%**
Null model	0.200	0.947	71.838	1.000	
ESRD etiology	0.200	0.013	0.055	0.014	0.0245
Dialysis duration	0.200	0.039	0.161	0.041	0.0064
ESRD etiology + Dialysis duration	0.200	0.0006	0.002	0.0006	0.9045
ESRD etiology + Dialysis duration + ESRD etiology * Dialysis duration	0.200	0.00005	0.00004	0.0001	0.6705

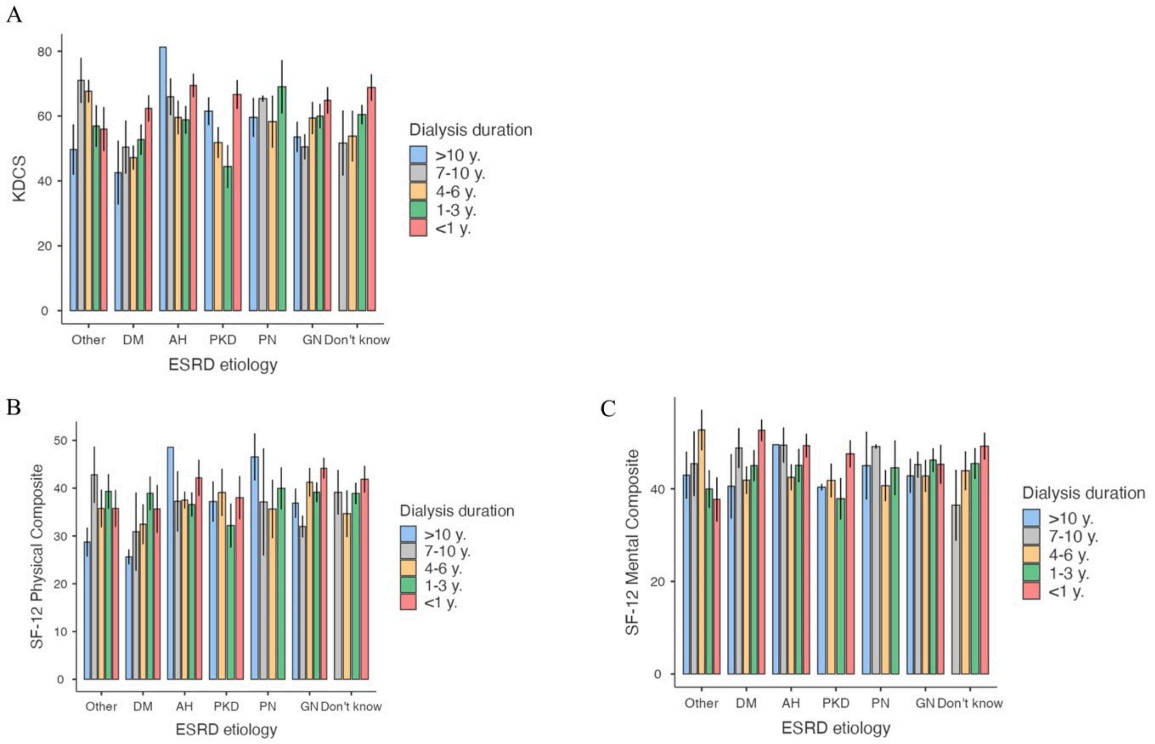

Suppl. Figure S1. KDCS, SF-12 Physical, and SF-12 Mental Composite scores by ESRD etiology and dialysis duration.

Distribution of (A) Kidney Disease Component Summary (KDCS) score, (B) SF-12 Physical Composite score, and (C) SF-12 Mental Composite score according to end-stage renal disease (ESRD) etiology and dialysis duration. ESRD etiologies include diabetes mellitus (DM), arterial hypertension (AH), polycystic kidney disease (PKD), pyelonephritis (PN), glomerulonephritis (GN), other causes, and unknown etiology ("Don't know"). Bars represent mean scores with standard error. Dialysis duration groups: <1 year, 1–3 years, 4–6 years, 7–10 years, and >10 years.
